# Production and characterization of miro- and nano-features in biomedical alumina and zirconia ceramics using a tape casting route

**DOI:** 10.1007/s10856-012-4635-1

**Published:** 2012-04-20

**Authors:** Maciej Domanski, Louis Winnubst, Regina Luttge, Edwin Lamers, X. Frank Walboomers, John Jansen, Han Gardeniers

**Affiliations:** 1 Mesoscale Chemical Systems, MESA+ Institute for Nanotechnology, University of Twente, P.O. Box 217, 7500 AE Enschede, The Netherlands; 2 Inorganic Membranes, MESA+ Institute for Nanotechnology, University of Twente, P.O. Box 217, 7500 AE Enschede, The Netherlands; 3 Department of Biomaterials, Radboud University Nijmegen Medical Centre, P.O. Box 9101, 6500 HB Nijmegen, The Netherlands

## Abstract

A process of micromolding, delivering micro- and nanopatterned ceramic surfaces for biomaterial applications is described in this work. To create the desired structures, tape casting of ceramic slurries on microfabricated silicon mold was used. Several tape casting slurry compositions were tested to evaluate the feasibility of transferring micro- and nano-features from silicon molds. Used ceramics were alumina (α-Al_2_O_3_) and yttria stabilized zirconia. Three types of polymeric binders for the green tape (PVB, PES, and PVP) were investigated using three different solvents (ethanol, *n*-methyl-pyrrolidone, water). Well-defined features in shapes of wells with diameters down to 2.4 μm and a depth of 10 μm and pillars with diameters down to 1.7 μm and a height of 3 μm were obtained. Morphology, grain size and porosity of the sintered bodies were characterized. Finally fibroblast cells were cultured on the surfaces in order to observe their morphology under influence of the microstructured surfaces.

## Introduction

Historically, ceramic biomaterials, such as alumina and zirconia, were anticipated to function as an inert material in the body. Nowadays the emphasis is more on applying these materials to create a bioactive scaffold that stimulates the construction of a well-functioning bone-implant interface [1–4]. It is often stated that a specific combination of micro- and nanotopography is required to stimulate osteoinduction or to cause mechanisms like mechanical anchoring of implant in the body, stimulate cells to produce a higher density of focal adhesions [5–7] or even to present an antibacterial effect [8]. Naturally occurring sub-micrometer range stochastic surface features on ceramics emerge from the intrinsic grainy nature of this group of materials. In addition to this “natural” nanostructure, in this study we will define micro-pillars, micro-wells and sub-micron sized ridges and grooves prepared by tape casting using microfabricated silicon as casting molds. Ceramic materials used here were yttria stabilized zirconia (YSZ) and alumina (α-Al_2_O_3_), which have a long history as implantation materials, both as a bulk material or in form of coatings [9, 10]. Such inert oxide ceramics are generally used in applications where good mechanical and tribological characteristics, that are unique for this group of materials, are required [1, 2, 9–11].

In fabrication process, tuning the ceramic slurry parameters allows us to obtain the desired bioceramics morphology, porosity, mechanical strength and surface micro- and nanotopography [12–16]. We evaluated three types of typical tape casting slurries based on different binders and solvents, and five types of microfabricated silicon mold masters with different micro-feature dimensions (10, 5, 2, and 0.5 μm). The casting slurry recipes were adapted from respective tape casting methodologies [12], ceramic hollow fiber fabrication by a phase inversion method [13] and colloidal filtration methods [14]. The micro-features in the silicon molds consisted of pillar- or well-like structures arranged in a trigonal lattice. To evaluate the dimensional limits of pattern transfer, casting was also performed on submicrometer silicon features with ridge and groove shapes. After casting, the green tapes were either air-dried or solvent was exchanged with non-solvent. The resulting green tapes were sintered and characterized with scanning electron microscopy (SEM) and densitometry. Finally, the produced size-resolved surface topographies were used in biological experiments, from which it was concluded that fibroblast cells respond to surface topography by altering their morphology. Although our research focused on YSZ and α-Al_2_O_3_, which are well recognized ceramic materials in biomaterial applications, we would like to stress that any type of ceramic material can be structured utilizing the versatile method presented in this work.

## Materials and methods

### Mold masters for tape casting

Masters were microfabricated using UV lithography for feature dimensions of 10, 5, 2 μm or laser interference lithography (LIL) for 0.5 μm features. Subsequently the pattern was transferred into silicon using reactive ion etching. In brief the fabrication of mold masters using UV lithography was conducted as follows: a 100-mm, standard {100}, *p*-type, one side polished silicon wafer (Okmetic, Finland) was spin-coated with positive i-line photo resist (OiR 907/17, Fujifilm) and subsequently exposed through a prefabricated chromium-on-glass mask using UV optical lithography. The exposure dose was 34 mJ cm^−2^. The latent structure was developed in OPD 4262 developer for 60 s (Fujifilm). Following lithography and development, deep reactive ion etching (DRIE) with a Bosch-type process was conducted with SF_6_ plasma composition in etching mode and C_4_F_8_ plasma in passivation mode in an ICP source type RIE equipment at −10 °C using liquid nitrogen substrate holder cooling (Adixen AMS100SE, Alcatel).

For the LIL delivered structure a tri-layer resist system was spin-coated on a silicon wafer using OPTIcoat ST22^+^ equipment (Sister Semiconductor). The stack consisted of: a DUV30-J6 bottom antireflective coating (BARC, Brewer Science), positive photoresist (PEK-500 chemically amplified resist, Sumitomo Chemical) and an Aquatar-6A top antireflective coating (TARC, Brewer Science). It was found that configuration of layers of 13 nm BARC, 140 nm photoresist, and approximately 5 nm TARC gave optimum stability and a high structural resolution under ambient conditions. In the lithographic step, a fourth harmonic continuous-wave yttrium aluminum garnet laser MBD 266 system (Coherent Inc., USA) with a wavelength of 266 nm was used as the coherent light source. A Lloyd’s mirror interference setup was utilized as an interference pattern generator creating latent ridge and groove pattern. Exposures of the resist were done with a dose of 4.5 mJ cm^−2^. A post-exposure bake was performed for 90 s at 105 °C. After lithography, latent resist patterns were manually developed for 45 s in 75 % v/v OPD 4262 in water (Fuji-film Electronic Materials). The first etching step, removing the BARC, was conducted in an oxygen plasma applying 280 W power, 1 Pa pressure 8 sccm O_2_ gas flow, and 18 s etching time. After oxygen plasma step, the system was switched to a SF_6_:O_2_ plasma composition and the silicon etching process was continued in standard RIE mode. The etching was carried out in a PlasmaTherm 790 (Unaxis) parallel plate etcher at 10 °C using substrate stage water cooling.

After etching the silicon molds were cleaned in fuming nitric acid for 10 min in order to remove residues of resist. Few types of anti-adhesion coatings on silicon molds were fabricated. Molds were sputtered with a thin layer of one of the materials: gold, titanium, chromium or aluminum (below 200 nm) using an argon plasma sputtering system with 200 W RF power (Sputterke, TCO) or a thin layer of carbon fluorides was deposited using plasma enhanced chemical vapor deposition (PECVD) using C_4_F_8_ (Adixen 100 SE, Alcatel).

### Preparation of ceramic slurries

The starting ceramic powders were either α-Al_2_O_3_ (Sumitomo AKP 30, particle size range 0.3–0.5 μm, surface area typically 5–10 m^2^ g^−1^) or YSZ (Tosoh Zirconia, TZ-8YS, particle size range 0.05–0.08 μm, surface area typically 5–9 m^2^ g^−1^). Three types of tape casting slurries were prepared: water based (referred to as PVP–H_2_O), ethanol based (referred to as PVB–EtOH) and *n*-methylpyrrolidone based (referred to as PES–NMP). The slurry compositions are summarized in Table 1.

**Table 1:** Composition of the tape casting slurries used for micromolding (weight%)

Composition	PVB–ethanol based slurry (PVB–EtOH)		PVP–water based slurry		PES–*n*-methyl-pyrrolidone based slurry (PES–NMP)	
Viscosity of slurry	~2,500 mPa		~6,000 mPa		~9,500 mPa	
Ceramic powder	α-Al_2_O_3_	42.9 %	α-Al_2_O_3_	46.3 %	8 mol% Y_2_O_3_ stabilized ZrO_2_	50.7 %
Binder	Polyvinyl butyral, PVB^b^	6.4 %	Polyvinylpyrrolidone (PVP)	4.5 %	Polyethersulfone PES^b^	4.9 %
Solvent	Ethanol	46.6 %	0.02 M nitric acid in H_2_O	46.2 %	*n*-methyl-2-pyrrolidone (NMP)	44.4 %
Dispersant	Solsperse 20000^a^	0.8 %	Disperbyk 194^c^	3 %		
Plasticizer	Butyl benzyl phthalate^b^	2.8 %				
Dispersant	Menhaden fish oil^a^	0.5 %				

^a^The Lubrizol Corporation, USA, ^b^ Tapecasting Warehouse, Inc., USA, ^c^ BYK-Chemie GmbH, Germany

#### Organic solvent based slurries

For preparation of ethanol and NMP based tape casting slurries two types of polymeric binders were used: PVB and PES, respectively (polyvinyl butyral, Tapecasting Warehouse INC and polyethersulfone, Goodfellow Cambridge Limited). First step of slurry preparation consisted of dissolving oven-dried polymers (50 °C/24 h). For PVB 99.9 % ethanol and for PES 99.9 % *n*-methylpyrrolidone (Sigma Aldrich) were used as the solvents. The polymer–solvent mixtures were stirred on a roller bench during 3 h in a 1 liter poly-ethylene bottle together with 1 cm diameter alumina balls. Next, the tape casting additives as given in Table 1 were added. Additives used in PVB–EtOH based slurry were: Menhaden Fish Oil and Butyl Benzyl Phthalate as plasticizer (Richard E. Mistler, Inc.) and Solsperse 20000 as dispersant (The Lubrizol Corporation). For the PES–NMP based slurry no dispersants or plasticizers were used. The solutions were mixed for one more hour. Oven dried (60 °C, 24 h) alumina or zirconia powders were added to the polymer–solvent solutions and the slurries were mixed on a roller bench for another 24 h. Finally, slurries were degassed in ultrasonic bath for 20 min.

#### Water based slurry

To prepare the water based slurry, the alumina ceramic powder was dispersed in 0.02 M aqueous nitric acid solution. The powder to solvent ratio was 1:1 (w/w). Next, the suspension was sonicated using a horn sonicator for 7 min (S-250, Branson Ultrasonic). Finally polyvinylpyrrolidone polymer (PVP, Aldrich, *M*_w_ = 1,300,000) and Disperbyk-194 (BYK-Chemie GmbH) dispersant were added and the suspension was mixed for 4 h on a roller bench.

### Tape casting on silicon masters and sintering

A thin (1–3 mm) layer of ceramic slurry was deposited on the silicon master by using a doctor’s blade. Next, depending on the slurry type, green tapes were slowly air dried in ethanol-vapor rich environment (for PVB–Ethanol based slurry) or in moist air (for PVP–water slurry), obtained by closing the sample together with a Petri dish filled with either ethanol or water in a 10 × 10 × 5 cm^3^ polymer box with a 0.5 × 0.5 cm^2^ hole in the top that caused slow diffusion-driven air–solvent exchange. After drying the green tapes were manually separated from the masters. The PES–NMP based green tape was prepared using a phase inversion method. The slurry was casted on the mold master and subsequently immersed in water to cause liquid–solid phase separation. This solvent/non-solvent exchange in the slurry causes solidification of PES [17]. Finally the samples were manually removed from the master, and dried for 72 h. The dried green tapes were cut in 5 × 5-mm^2^ pieces and sintered in a tube furnace (Vectstar, type 6Z) at 1,500 °C. Sintering profiles were designed as follows: heating at 1 °C per minute to 400 °C, binder burn-out at 400 °C for 1 h, heating up to 1,500 °C, sintering for 4 h, and finally cooling down to room temperature at 3 °C per minute.

### Density and grain size measurement

For the sintered ceramics grain size analysis was performed, utilizing the linear intercept method for analysis of SEM scans. Sintered specimens were polished using diamond polishing media (Cameo method, LamPlan). Next specimens were thermally etched in a tube furnace by heating up to 1,230 °C and keeping the specimens at that temperature for 45 min in order to reveal the grains. After this treatment both zirconia and alumina specimen were sputter coated with a thin gold layer and investigated by SEM. Image analysis utilizing ImageJ software was used to measure the average grain size with the line intercept method (Mendelson method, Fig. 5) [18]. Density of the sintered ceramics was measured with the Archimedes’ method [19] in mercury using a laboratory scale (Mettler Toledo PB1502S). The detailed morphology of specimens was investigated with SEM. All SEM scans included in this work were made on Jeol JSM-5610LV, Jeol 6310 SEM or Philips XL 30 ESEM-FEG electron microscopes.

### Cell culture study

Rat dermal fibroblasts (RDF) were obtained from the ventral skin of male Wistar rats as described by Freshney [20]. Cells were cultured in α-MEM medium (Invitrogen) supplemented with 10 % Fetal Calf serum (FCS) and gentamicin (50 μg ml^−1^). Cell culture experiments were performed at the 6 or 7th culture passage. Before transferring the cells to the substrates, cells were detached from the culture flask using trypsin/EDTA (0.25 % w/v trypsin/0.02 % EDTA) (Invitrogen) and concentrated by centrifugation at 1,500 rpm for 5 min. Subsequently, cells were resuspended in the culture medium, quantified using a Coulter^®^ counter (Beckman Coulter Inc.) and seeded at a density of 1 × 10^4^ cells cm^−2^. Fibroblast cell culture assays were performed on well- and pillar-like structures of α-alumina substrates acquired from PVB–EtOH slurries in the time of 2 days of seeding. Thereafter cells were fixed in 2 % (w/v) glutaraldehyde in 0.1 M sodium-cacodylate, dehydrated in a graded series of ethanol (from 70 up to 100 %), and dried to air in tetramethylsilane. The substrates were sputter coated with gold and observed using a Jeol 6310 SEM.

## Results and discussion

### Ceramics fabrication

**Fig. 1:**
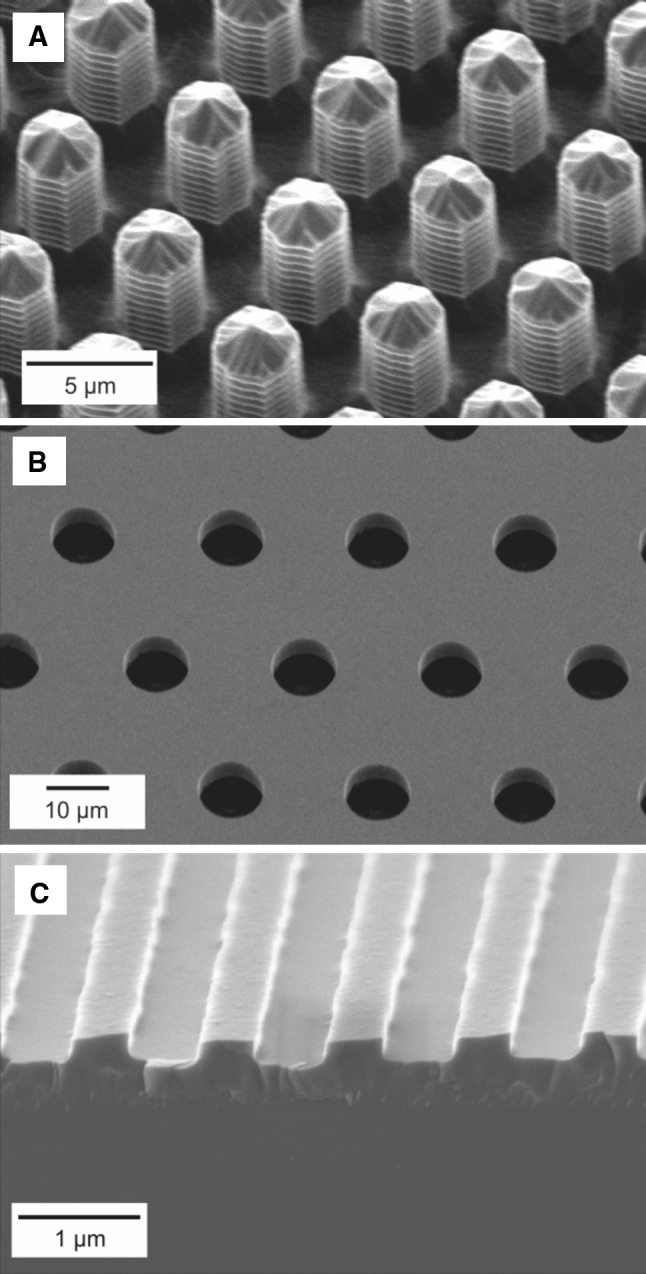
Scanning electron micrographs of silicon mold masters fabricated with UV lithography (**a**, **b**), LIL (**c**) lithography and subsequent RIE. **a** 2 μm pillar structure in silicon. **b** 10 μm well structure etched in silicon. **c** 0.5 μm ridges in silicon

SEM micrographs of etched silicon molds of UV lithography delivered well- and pillar-like structures and the LIL delivered ridge and groove structures are presented in Fig. 1. It is clearly visible that uniform patterns can be obtained with the microfabrication techniques used in this work. Slightly negative tapers in etched pillars and wells obtained by the DRIE process can be of an additional advantage in flawless mold release; however, the influence of this was not studied in this work. In order to facilitate easy demolding an anti-adhesion layer was deposited on the mold surface. The role of the anti-adhesion layer was to facilitate demolding by lowering adhesion of binder polymer to the silicon microstructure. We have tested five different surface coatings: sputtered gold, titanium, chromium, alumina or PECVD of C_4_F_8_. In order to choose the method facilitating the most complete release of green tape from the mold, all surfaces coatings were evaluated with all water and organic solvent slurries. For the phase inversion system utilizing NMP-based the anti-sticking coating of carbon fluorides gave the best results and for water and ethanol based slurries gold was the most satisfying choice, guaranteeing the most complete surface features replication. The thickness of the metal coating was below 200 nm and did not significantly change the dimensions of the surface features.

**Fig. 2:**
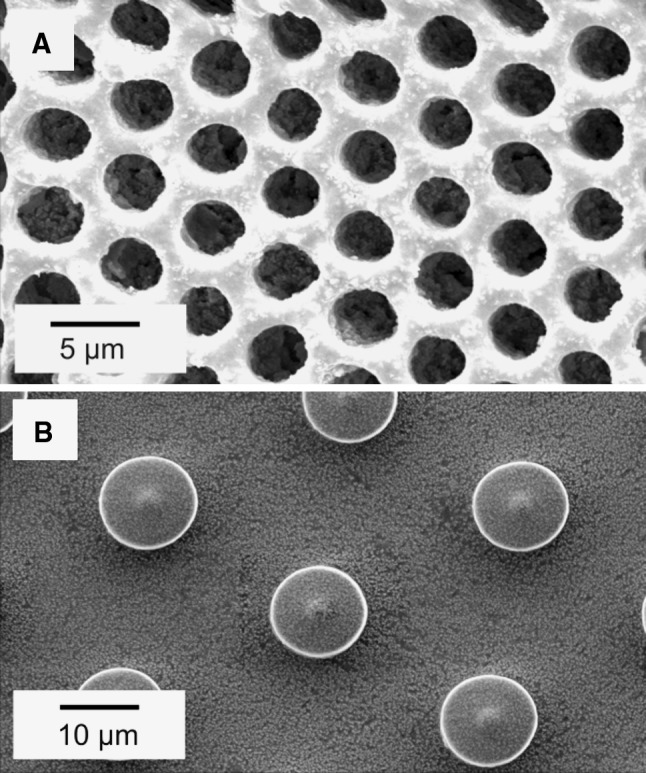
Green tapes with visible polymer-ceramic composite before sintering. **a** 2 μm well structure created in ZrO_2_ with PES–NMP slurry. **b** 10 μm pillar structure created in Al_2_O_3_ with PVP–H_2_O slurry

After tape casting and demolding, the green tapes were examined with SEM. In the acquired micrographs the dispersed particles of ceramics suspended in the polymer matrix could be observed as bright dots in contrast to more electron adsorbing and thus darker polymer matrix (Fig. 2). No large agglomerates of ceramic particles were observed for all types of green tapes. This suggests that the dispersion stabilization by either polymer additives or pH adjustment was successful. The PVB–EtOH and PES–NMP based slurries gave robust easy to handle green tapes, facilitating further shaping (i.e., in bone implant shape) whereas PVP–H_2_O based slurry gave mechanically brittle green tapes. For all casting methods used the demolding process results in up to 99 % of complete and defect-free green tapes, for both pillars and wells.

**Fig. 3:**
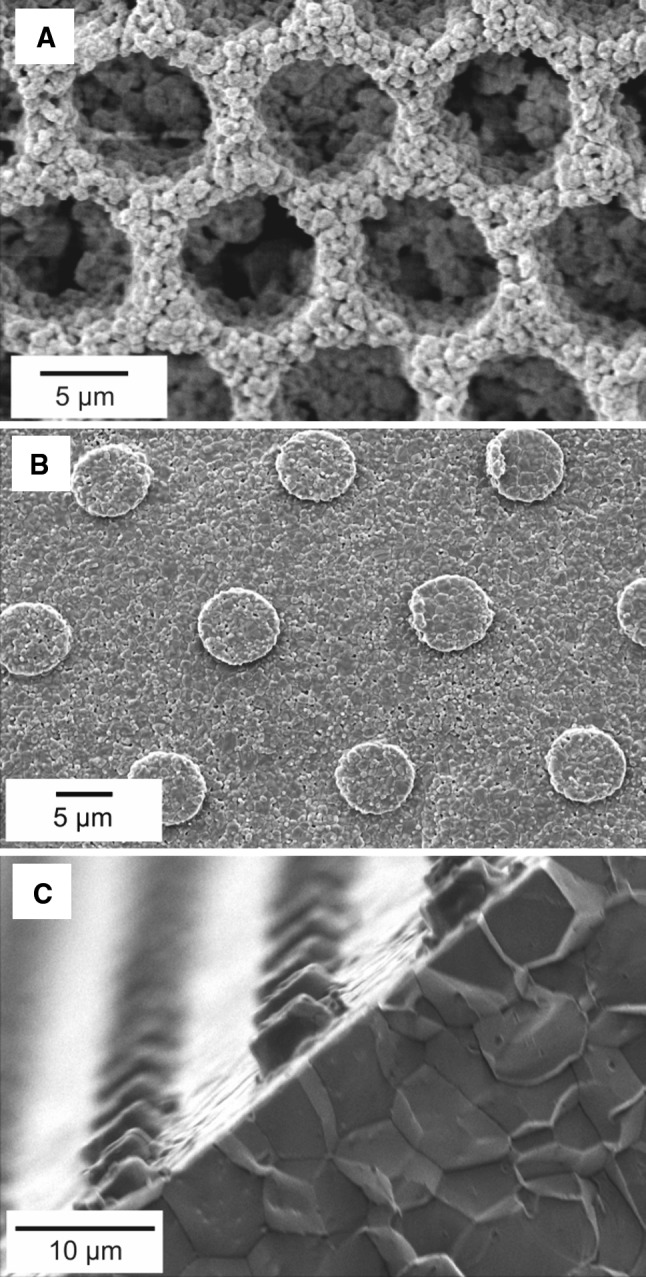
Sintered in 1,500 °C ceramic bodies. **a** 6 μm well structure created in ZrO_2_ with PES–NMP slurry. **b** 8 μm well structure created in Al_2_O_3_ with PVB–EtOH slurry. **c** Al_2_O_3_–PVP–H_2_O slurry delivered structures

**Fig. 4:**
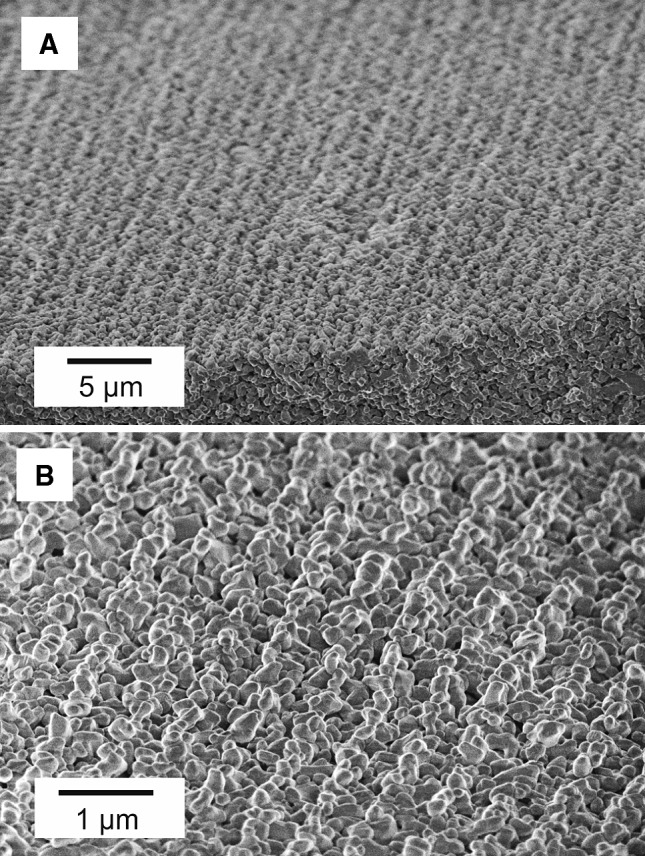
Surface of nanopatterned sintered zirconia (PES–NMP slurry) shaped with 0.5 μm silicon mold master delivered by LIL and RIE, barely visible ridges diminish due to the grain size

As a result of sintering process, different structural shrinkage occurred with different ceramic formulations. In general the most complete replication copies were obtained with Al_2_O_3_ PVB–EtOH slurry. For this formulation the shrinking during sintering was lowest and the critical dimension and shape definition of micro-features changed the least. After sintering, the smallest transferred features from UV delivered molds were approximately 1.7 μm-diameter pillars (master dimension was 1.9 μm), and 3.2 μm-diameter wells (master dimension was 4.8 μm). These features were obtained from all three tape-casting slurries. For the LIL delivered master with 500 nm wide ridges only the PES–NMP based slurry containing zirconia ceramics resulted in a structured surface, however, the replication quality was very low (Fig. 4).

**Fig. 5:**
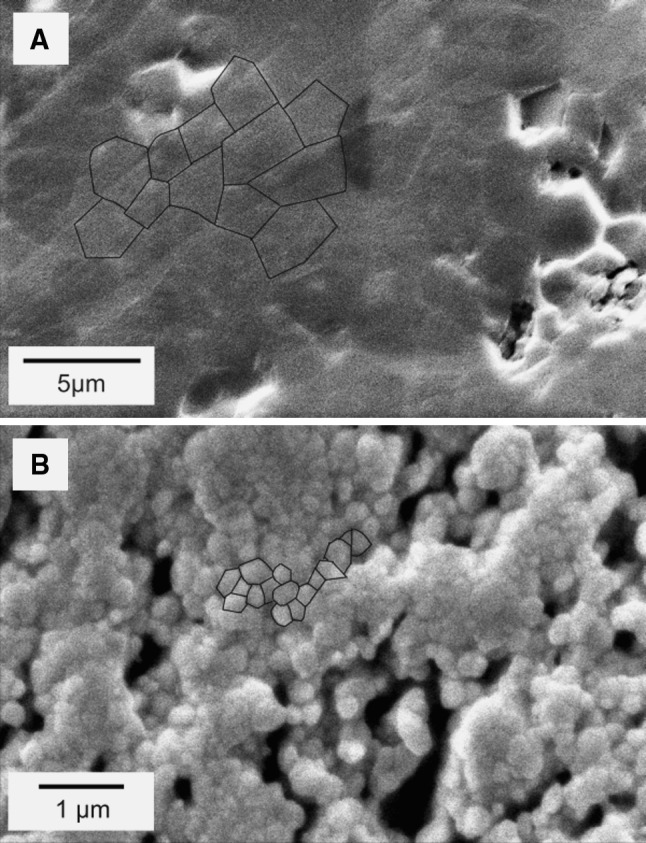
The ceramic samples after sintering. Polishing and temperature etching were used to unveil grain boundaries. **a** Surface with outlined grain of alumina. **b** Surface with outlined grain of zirconia

For both Al_2_O_3_ and ZrO_2_ based ceramics fabricated with PVB–EtOH and PES–NMP slurries, the grain structure on the surface of sintered specimens is clearly visible (Fig. 3a, b). In case of Al_2_O_3_ specimens delivered with PVP–H_2_O slurry, this structure can only be observed in bulk material (Fig. 3c) and cannot clearly be observed on the surface of sintered material which appears to have a non-porous structure. The density of sintered bodies was measured with the Archimedes’ method and ranged from the lowest for the NMP–PES-derived system (88 %) via PVB–EtOH-derived (96 %) to the high-density PVP–H_2_O-derived system (99 %; relative values to theoretical density values of alumina and zirconia of 3.97 g cm^−3^ and 5.68 g cm^−3^, respectively). For the PVB–EtOH and PVP–H_2_O derived alumina ceramics significant grain growth can be observed, where the largest grains were obtained for the PVP–H_2_O based slurry. The initial Al_2_O_3_ powder had a grain size of 0.3–0.5 μm. In the sintered body grains have grown to approximately 2.4 μm in diameter for the PVP–H_2_O slurry and 1.3 μm in the green tapes obtained with the PVB–EtOH slurry. In case of zirconia powder the growth was also significant, the measured grain size in the sintered body was 0.48 μm (0.05–0.08 μm initially). Grain growth during sintering occurred to be the main limiting factor for copying structures with critical dimensions smaller than 6 × *D*_grain_ (Fig. 4). Morphology measurements are summarized in Table 2.

**Table 2:** Parameters of green tape and sintered ceramics

Composition	Volume ratios in green state		Density of sintered body	Grain size in sintered body	Shrinkage of sintered ceramic (*D*_mold_/*D*_ceramics_)
	Ceramics	Binder			
Al_2_O_3_ PVB–EtOH	70.4 %	29.6 %	96 %	1.3 μm	0.92
Al_2_O_3_ PVP–H_2_O	76.9 %	23.0 %	99 %	2.4 μm	0.77
ZrO_2_ PES–NMP	69.4 %	30.6 %	88 %	0.48 μm	0.66

### Cell culturing tests and results

**Fig. 6:**
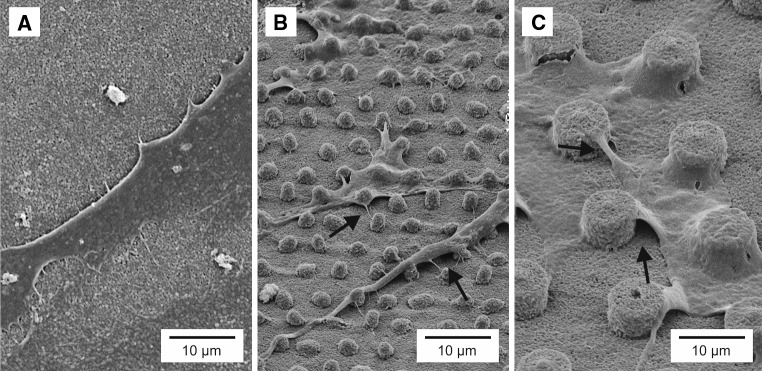
SEM micrographs of fibroblasts (RDF) cultured on micropatterned substrates: **a** Flat alumina, **b** 1.7 μm pillars, **c** 8.6 μm pillars. The *arrows* indicate alternations in morphology of fibroblast caused by microstructure

Rat dermal fibroblasts were cultured on alumina samples delivered from Al_2_O_3_ PVB–EtOH slurry. On flat alumina controls, fibroblasts were well-spread and formed normal spindle and multipolar cell morphologies with short filopodia. However, in contrast to cells cultured on standard cell culture polystyrene substrates (reference material, not shown), many focal contacts were visible at the cell edges (Fig. 6a) specifically adhering to the nanorough grain boundaries and small pores on the ceramic substrate surface. Fibroblast cells that had been cultured on the micropillar substrates were changing their morphology by stretching on the pillars. These cells also exhibited large filopodial extensions. Cell bodies covered the pillars and descended down to the substrate surface around the pillars. The larger cell extensions clearly appeared to be guided by the pillars (Fig. 6b, c). No apparent differences in cell morphology were observed between all structural dimensions (1.7, 4.6, and 8.6 μm) on which the cells were cultured on. Osteoblasts and fibroblasts have already been shown to respond to nanoroughness by increased filopodia formation and increased adhesive strength [22–24]. In accordance with these studies, the number of filopodia also appeared to increase on the currently tested micro-features structured alumina substrates. The observed phenomenon associated with such an event is a localization of *F*-actin, stress fibers and focal adhesions, marking the site of the discontinuities, edges, underlying the cell. The full effect of this phenomenon on an individual cell phenotype and differentiation pathway has yet to be deciphered [22–24].

## Conclusions

In this work pattern transfer into ceramics was evaluated by tape casting from microfabricated silicon molds. These microfabricated ceramic surface structures could have application as a bio-active material. The influence of surface features on cells was evaluated in fibroblast culture and morphology study. Here, we have used silicon molds with pillar- and well-like microstructures in a trigonal lattice arrangement as well as submicron sized ridge and grooves. Silicon mold microfabrication was based on photolithography (UV and LIL) and silicon dry reactive ion etching. This process provides a large flexibility in the design of microstructural layouts of surface features. Tape casting of the ceramic slurry on the surface of the silicon mold was used to replicate the silicon patterns in ceramics. It was found that all three investigated slurry systems (Al_2_O_3_ PVB–EtOH, Al_2_O_3_ PVP–H_2_O, ZrO_2_ NMP–PES) were able to replicate well and pillar structures from the mold master down to 1.7 μm and zirconia based slurry also ridge and groove structures in submicron size range. The sintered ceramics from different casting systems resulted in different densities and bulk microstructure. Initial grain size and grain growth during sintering were the main factors limiting small feature transfer from mold to final product. Using fine ceramic powders and preventing grain growth during sintering could lead to more uniform and well defined microstructures. The Al_2_O_3_ PVP–H_2_O slurry gave the ceramic bodies with the highest density and the ZrO_2_ NMP–PES gave the structures with the smallest grain size (note that for zirconia the initial grain size was also smaller). Finally to evaluate potential biomaterial–cell interactions, RDF were cultured on the microstructures delivered using Al_2_O_3_ PVP–H_2_O slurry which showed best structure replication performance. The findings from these cell cultures suggest that cells mainly react to micro pillars and wells by alterations in cell morphology and filopodia sensing. The cells recognize the patterns and respond by adapting a more stretched morphology including the formation of filopodia showing intimate contact with the surface features. The fact that alumina substrates possess an inherent nanoroughness can also influence cell behavior, as it was observed for combinations of other non-ceramic nano and microstructures [21]. It is recognized in literature as a “feature edge effect” and is also responsible for mechanisms like topographically induced cell guidance [4, 5],. Finally we conclude that tape on microfabricated silicon molds can be used to deliver microtopographies on ceramic surfaces with high reproducibility and good critical dimension control for biomaterial applications.
